# Cloud- and Fog-Integrated Smart Grid Model for Efficient Resource Utilisation

**DOI:** 10.3390/s21237846

**Published:** 2021-11-25

**Authors:** Junaid Akram, Arsalan Tahir, Hafiz Suliman Munawar, Awais Akram, Abbas Z. Kouzani, M A Parvez Mahmud

**Affiliations:** 1 School of Computer Science, The University of Sydney, Camperdown, NSW 2006, Australia; 2 Department of Computer Science, Superior University, Lahore 54000, Pakistan; 3 Research Center for Modelling and Simulation, National University of Sciences and Technology (NUST), Islamabad 44000, Pakistan; atahir.mscse17@rcms.nust.edu.pk; 4 School of Built Environment, University of New South Wales, Kensington, NSW 2052, Australia; h.munawar@unsw.edu.au; 5 Department of Computer Science, COMSATS University Islamabad, Vehari 61100, Pakistan; awaisakram1212@gmail.com; 6 School of Engineering, Deakin University, Burwood, VIC 3125, Australia; abbas.kouzani@deakin.edu.au (A.Z.K.); m.a.mahmud@deakin.edu.au (M.A.P.M.)

**Keywords:** smart grid, fog computing, binary particle swarm optimisation, cloud computing, makespan minimisation

## Abstract

The smart grid (SG) is a contemporary electrical network that enhances the network’s performance, reliability, stability, and energy efficiency. The integration of cloud and fog computing with SG can increase its efficiency. The combination of SG with cloud computing enhances resource allocation. To minimise the burden on the Cloud and optimise resource allocation, the concept of fog computing integration with cloud computing is presented. Fog has three essential functionalities: location awareness, low latency, and mobility. We offer a cloud and fog-based architecture for information management in this study. By allocating virtual machines using a load-balancing mechanism, fog computing makes the system more efficient (VMs). We proposed a novel approach based on binary particle swarm optimisation with inertia weight adjusted using simulated annealing. The technique is named BPSOSA. Inertia weight is an important factor in BPSOSA which adjusts the size of the search space for finding the optimal solution. The BPSOSA technique is compared against the round robin, odds algorithm, and ant colony optimisation. In terms of response time, BPSOSA outperforms round robin, odds algorithm, and ant colony optimisation by 53.99 ms, 82.08 ms, and 81.58 ms, respectively. In terms of processing time, BPSOSA outperforms round robin, odds algorithm, and ant colony optimisation by 52.94 ms, 81.20 ms, and 80.56 ms, respectively. Compared to BPSOSA, ant colony optimisation has slightly better cost efficiency, however, the difference is insignificant.

## 1. Introduction

The smart grid (SG) controls energy distribution and allows Internet of Things (IoT) devices to communicate with one another. End users and service providers can engage in two ways with the SG, allowing them to track their energy use and price [1]. Because of the vital nature of the SG’s services and the large number of customers using them, rapid response and processing times are critical. To handle a huge number of requests generated by a variety of devices such as a smart metre, cloud data must be handled, processed, and stored [2]. The Cloud’s evolution has reduced the requirements for substantial computing capacity at the device level [3]. The smart metre, which is permanently saved on the Cloud, provides individual data and energy use statistics to the smart grid. The Cloud, when combined with IoT connectivity, offers a wide range of services and allows for intelligent data management [4].

The rapid development of IoT devices has greatly increased the Cloud’s computing burden, resulting in longer response and processing times, which might be important for time-sensitive applications. When sending data to the Cloud, SG ensures privacy [5]. As the number of cloud end users grows, it becomes more difficult to manage requests, and load balancing challenges occur [6]. The fog computing layer is established between the Cloud and the end users to reduce the effect of an increased processing time and manage massive requests from end users.

Cloud computing is a centralised system that uses shared computers rather than decentralised servers to perform computations. The Cloud delivers software as a service (SaaS), platform as a service (PaaS), and infrastructure as a service (IaaS) as well as other services [7,8,9]. Fog computing is a type of cloud computing that improves system performance. Fog directly interacts with end users and delivers cloud-like computing and storage services [10,11,12,13]. Fog also has a number of useful functions, such as location recognition and quick reaction time [14,15]. Fog computing enhances the validity of SG while lowering the cost of processing and reaction time [16,17]. End-user inquiries are sent to a cloud-based fog platform where they are routed to micro-grid (MG) services. Customers send requests to Fog, which locates the nearest MG and contacts the micro-grid controller. Processing and reaction times are accelerated and the overall performance is enhanced thanks to Fog [18,19].

We present an integrated SG model with Cloud and Fog in this study. In the proposed approach, the fog server receives electricity requests from end users and passes them on to the MG. End-user queries to Fog are managed using a dynamic service broker policy. Virtual machines (VMs) are present in each fog node. The necessary computations are handled by virtual machines. A ’Load Balancer’ mechanism is included in each fog node. The fog load is distributed over several virtual machines by the load balancer, which minimises the complexity of fog servers.

In recent years, Internet of Things devices have seen a rapid growth in all domains of modern-day life. The number of IoT devices is expected to grow even more rapidly in the future. Due to this trend, IoT devices associated with SG have tremendously increased the computation requirement of cloud servers [20,21,22,23]. To mitigate the effect of large computation requirements, the concept of centralised cloud- and fog-based platform is presented in [24]. The increased number of IoT devices not only increased computation requirements but also caused an increase in the response time [25,26,27]. Moreover, data storage in the Cloud has also become a critical issue. To manage this, the authors in [28,29,30] presented the concept of fog computing. Fog computing is a layer of processing and communication nodes that relieves the Cloud of the burden of multitasking while managing large quantities of user requests. Demand overload is a problem in cloud- and fog-based smart grids due to the unpredictable receipt of requests. Due to the sporadic utilisation of virtual machines, some resources are frequently and highly utilised while others are idle. Load balancing is a network distribution strategy that distributes a large number of virtual machines or CPUs. This aids in the achievement of balanced use which enhances performance while reducing the reaction time. As a result, efficiently balancing the load among the resources is a critical challenge. The main problem with fog computing is to competently manage incoming user requests with quick response times while preventing resource under-utilisation at the same time. By distributing requests to virtual machines, several methods are utilised to fulfil client requests with the shortest response time possible. Because each virtual machine in the Fog accomplishes the same amount of work throughout, load balancing is critical, lowering the reaction time and improving the total system output. By dynamically shifting the load to VMs or nodes, a machine’s usage may be balanced. Different load-balancing strategies have been outlined in the literature for the handling of user requests in the Cloud. This study proposes a novel load balancing method based on the aforementioned notion.

The application of computational intelligence (CI) approaches to improve the performance of systems for multi-objective, multi-level optimisation problems in cloud and fog computing is the major emphasis of this study. In general, optimisation issues are divided into two categories based on the variables involved: combinatorial and continuous optimisation problems, respectively, for discrete and continuous variables. Combinatorial optimisation problems are addressed in this article, and the results are presented and analysed. The following are the paper’s key contributions:

- The implementation and design of the particle swarm optimisation method;
- The use of more than one optimisation algorithm together to form hybrid versions such as the use of the simulated annealing (SA) method to enhance the performance of binary particle swarm optimisation (BPSO) and the quality of its solutions;
- Two optimisation methods are applied to cloud scheduling—in order to decrease the response time and execution time of tasks on the Cloud and Fog.

The rest of the paper is divided into the following sections: Section 2 explains the smart grid and the three architectures that the smart grid is built on. Section 3 discusses the system model, problem formulation, and proposed load-balancing technique. Section 4 discusses the results in detail. Section 5 summarises this work and presents the conclusion of the work.

## 2. Literature Survey

**Figure 1:**
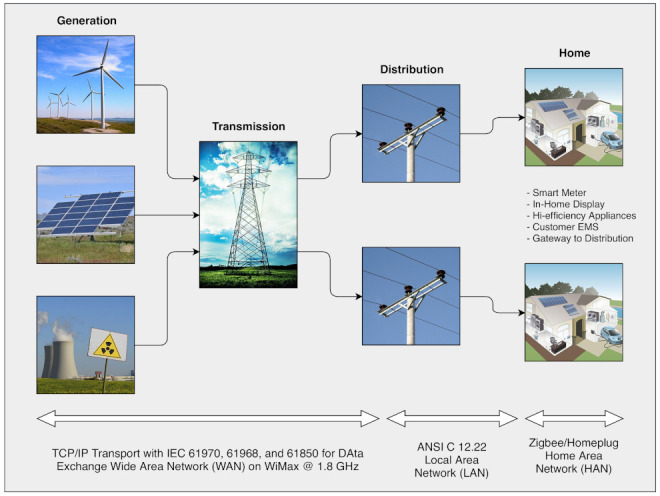
Overview of a typical smart grid architecture.

Smart grids are one type of cyber physical system. They are power electricity systems or networks. They generate electric power and transmit this power to customers such as factories [31,32,33,34]. Smart grids are one of the largest interconnected networks around the world. A failure in one part of smart grid can cause failures to the whole network of the smart grid. An overview of a typical smart grid architecture is shown in Figure 1. Examples of smart grids can be wind power such as wind turbines, which produce electricity power through turbines [35,36,37]. Wind power is one of the renewable energy forms that reduce the production of carbon dioxide [38,39,40].

Studies have shown that demand for smart grid consumption is rapidly increasing. The reason forb this is that it increases the efficiency of the supply chain. Consumers tend to use it in an effective manner [41,42]. The other reason for using smart grids is that they use less fossil fuels energies. Fossil fuel energies produce more CO_2_ and pollution and cause an increase in global warming [43,44]. Smart grids are based on renewable energy systems. They do not produce CO_2_, which is harmful for the environment. We can also see this electricity consumption in transportation. Some people tend to use electric cars, which use electricity power instead of gasoline. Some buses are hybrid buses, which use electric power [45,46,47].

The benefits of smart grids can be that they can reduce the peak load demand or optimise it. This leads to a reduced generation of electricity power [48,49]. Another benefit is that smart grids can increase energy efficiency because they can make customers more involved in the electricity usage [50,51]. However, in addition to the benefits of using smart grids, there are some disadvantages such as security. Attackers can access the smart grid network and hack into it to retrieve information or damage the system [50,52].

The security of smart grids is an important matter as it involves critical user data. If security is not taken care of, it can compromise the overall efficiency of the system. The leakage of consumer data or the compromise of the system can lead to huge costs and efforts to recover them and have further ramifications and economic impact [53,54]. Reliability can be one of the security challenges of smart grids. The other challenge can be quality of smart grids [55]. The main features of smart grids could be that the smart grid can provide smart meters for customers. Smart meters can measure the amount of use and price of use [56,57]. The smart meter provides security and therefore, the attacker might not be able to access it [58].

To provide solutions for cost-effectiveness in power grid systems and ways to deal with real-time management, numerous solutions have been presented. In accordance with already-presented architectures, three classes were found for these studies.

### 2.1. SG-Derived Architecture

A revised algorithm for energy management derived on the basis of the renewable energy sources (RES) concept (i.e., PV panels, wind turbine, hydropower systems etc.) and demand response was introduced by [59,60]. A smart grid-based architecture model includes photovoltaic panels, principles of demand response, the sharing of various resources for the provision of load, and state of charge. Despite the minimisation of the makespan and energy cost, there are some limitations to the model. The limitations are made effective by using Pareto optimisation [61,62] along with multi-objective genetic algorithm. Consumers observed optimal results after the application of these schemes [63]. Yoldas et al. in [64,65] discussed a review based on the revolutionary changes that occur in new power systems. This study is presented on the basis of challenges that take place due to the increase in the demands of electricity worldwide, global scale lacking in RESs integration, minimisation of carbon emission due to the inadequate cognizance of consumers, and the addition of communication technologies. These previously presented challenges brought the awareness to establish MGs (power panels for a smaller scale) which, due to the integration of technology and smart grid tools, are becoming an emerging domain [66,67]. Furthermore, the authors in [64] also shared talks about the MG along with its functionalities and the smart services in the smart grid environment.

Massive amounts of Big Data obtained from smart metering in distributed smart energy grids were accepted by the proposed architecture in [68], which allows for automated power commercial transactions between participants in a specialised marketplace. Values fluctuate in function of real-time supply and demand as well as the grid’s state. As a result of utilising the knowledge provided by the energy marketplace, all participating stakeholders may make the most out of their product while also contributing to the overall stability of the energy grid.

For commercial, industrial, and residential loads, Refs. [69,70] followed a genetic algorithm utilisation scheme and presented demand management in smart grids. In this system, only adaptable and fixed devices are considered. For minimising the average cost-to-peak ratio, the elastic load was rescheduled using a genetic algorithm. In the mobile appliances, after being rescheduled, there was a reduction observed in the average cost-to-peak ratio. Barbato et al. in [65,71] proposed a complete strategy for requested management using the infrastructure of smart grids to reduce the peak ratio for the customers. This study was based on the idea of using two different kinds of strategies, i.e., a hybrid strategy and completely distributed strategy. Other devices employ self-governing decision in the first type, but in the second type, i.e., the hybrid, the devices are scheduled based on the demand. By looking at these strategies in the grid-connected modes, the performances are discussed on the basis of the numerical evaluation. Following particular system constraints, an uncooperative game is used to model the system. After the evaluation, it was declared that there was a 55% minimisation in the peak formed. Appliances are selected to fulfil the peak average ratio, the comfort of the user, and the objectives of cost. Furthermore, smart grids’ demand response has been increasing with growth in smart devices. However, due to the issue of managing huge data and an increment in consumer involvement, it becomes computationally difficult. To deal with such issues of scalability, computation, and emergencies, an efficient and reliable system meeting the requirements is needed.

### 2.2. Fog- and Cloud-Derived Architectures

A great deal of attention has generated by the cloud computing paradigm in the literature [72,73]. It has been suggested that cloud computing devices are strongly associated with their architectural frameworks. In terms of the non-technical and technical challenges, the authors presented the distinctive points and concepts of cloud computing, also talking about the various future directions highlighted by Armbrust et al. in [74]. An approach supporting the content retrieval for the images that are encrypted without them being exposed by the cloud server was discussed by Xia et al. in [75,76]. To begin with, the feature vectors are used to extract the images. The pre-filter tables are then used to develop a locality hashing to enhance efficiency surfing. In addition, to secure the feature vectors, the k nearest neighbour (kNN) algorithm was applied for the protection mechanism. To protect the images, a standard stream cipher was used to apply the encryption mechanism. To prevent illegitimate image spreading, a protocol based on the watermark was proposed and the cloud server was used to add the watermark before transmitting images. In case a copy of an illegitimate image is achieved, then the extraction process based on the watermark is used to extract the user. This process is useful in promoting efficiency and protection.

A consumer model with a scheme based on the surfable encryption was developed using a ranked multi-keyword search. Fu et al. in [77,78] described this scheme as the purpose to provide security in the cloud computing environment. Xia et al. presented a similar technique for cloud computing updates in [51,79]. This technique is operational for the encryption of data in cloud computing. It assists in the insertion and deletion of documents and dynamically updates them. For query transmission and index creation, TF x DF models and vector-based models are collectively used. Greedy depth-first search algorithm and an index organisation—being tree-based—were used to intensify the efficiency of surfing. The kNN algorithm was used for the security of query vectors and indexes’ encryption. These algorithms depict the score for both query-generated and indexes vectors. For the insertion and deletion of documents, this scheme sub-linearly used the surfing time which enhances the scheme efficiency.

To resolve the problem of delay, latency, hourly requests, and response time, Bonomi et al. in [80] proposed an alternative fog computing paradigm. Fog computing provides us with solutions for reliability, delay, and time for processing [81]. Nonetheless, in both the emerging fog and cloud technologies, requests are randomly sent from consumers by any process. Problems such as overburden and congestion are seen due to a huge number of requests [82]. However, the unfair task assignment is linked to the overburden of the servers. Load imbalance is due to random task assignment which occurs due to overloaded and under-loaded processors [72]. Khiyaita et al. in [83] performed a review finding that the exploitation of future research obstacles in smart grids may present techniques such as load balancing in cloud computing. The purpose of load balancing is to transmit the load from overloaded to under-loaded processors. Coordination must be enabled by the system to meet with the consumers’ requests [84]. This may present congestion and it is also directed to create imbalance in the management of services. For service balancing, load balancing algorithms are required by cloud computing with minor modifications [85]. Various researchers are working to solve this issue, for instance, the authors in [86] presented a remedy to deal with the load-unbalancing problem. Various types of loads such as the network loads, memory, and computation are considered in this work. To balance all such work in cloud computing, various heuristic algorithms such as genetic algorithms, tabu search, and simulated annealing were considered [87].

Nikhit et al. in [88] discussed a hybrid ACHBDF (ant colony honey bee with dynamic feedback) algorithm for resource utilisation in cloud computing optimisation for a load balancing mechanism. This algorithm uses the feedback methodology of the dynamic time step utilising two run time scheduling collective schemes. For effective task scheduling, it also depends on the quality of honeybee algorithms and ACO. For the maintenance of the dynamic feedback method, load verification is performed by the support system before each iteration. Bitam et al. in [89] suggested an improved bees’ life algorithm (BLA)—a bio inspired algorithm for effectively assigning the tasks in the environment of fog computing. This study was based on fog node distribution. The main objective of this approach is to achieve a trade-off between storage-utilising fog nodes and the execution time of the CPU. The cost and response time for this scheme was also measured and compared with the previous genetic and PSO algorithms. Out-performance was observed in this case study.

### 2.3. SG with Cloud-Derived Architecture

Many scholars, such as Mohamed et al. in [90], presented a technique known as service-oriented middleware (SOM) on the usage of cloud–fog computing to manage and support smart grids for the effective anatomising of hindrances during the operation and development of the functionalities of smart cities based on fog and cloud computing. Smart city ware, also known as SOM, includes fog and cloud computing features. This service provides users with multiple functionalities along with parameters extracted by the SOM. This works to improve the services’ parameters and functionalities, as requested by the consumers in the smart city. Moreover, the problems in the management of resources are not considered. Reka et al. in [45] presented a smart grid data-based model on cloud computing which takes the distribution advantage of data management for ubiquitous access, data gathering, and parallel processing for information retrieval. For the effective load management of smart grids users, stochastic programming is present in cloud computing. The Gurobi utilizer in Matlab and GUI (designed interface) gives off results. The main idea behind this is to reduce energy requirements through the addition of hubs.

Moreover, Moghaddam et al. in [50] presented a demand response of the cloud-based architecture—namely the cloud-based demand response architecture. The demand responses are of two kinds: distributed demand and cloud-based demand response. These are utilised by two models such as the communication models and a demand response model. Enhanced bandwidth utilisation and convergence time is minimised by this study. Lastly, these models proved that communication costs can be increased through more consumer requests and the proposed system efficiency. In [55], the authors presented an enhanced smart grid electric vehicle based on the cloud environment scheduling data by public service stations. This secures communication between cloud platforms and smart grids. When using priority assignment algorithms, the waiting time is also considered. For the EV process, algorithms of two types are used, such as the random priority optimisation algorithms and calendar priority optimisation. For each EV user, the four types’ priorities are included. Moreover, this study only deals with the problems of EVs and declines the request of buildings or home management. Moreover, Gu et al. in [91] handled a cloud data centre for green scheduling. The purpose of this work was to ensure the trading of the energy along the power grid. The main objectives were reducing the carbon emission and energy cost. To cope with hazardous emission energy, renewable sources were used.

The brief overview of the above-discussed studies shows that the need for a reliable cloud- and fog-derived smart grids scheme was inevitable for the efficient management of resources that deal with utility activities and the consumer. Therefore, a scenario is considered where smart buildings and homes are requested by the users and servers are used to handle the requests. These are optimised by the servers in such a way to reduce the response time, the cost of the resources, and the processing time.

## 3. System Model

**Figure 2:**
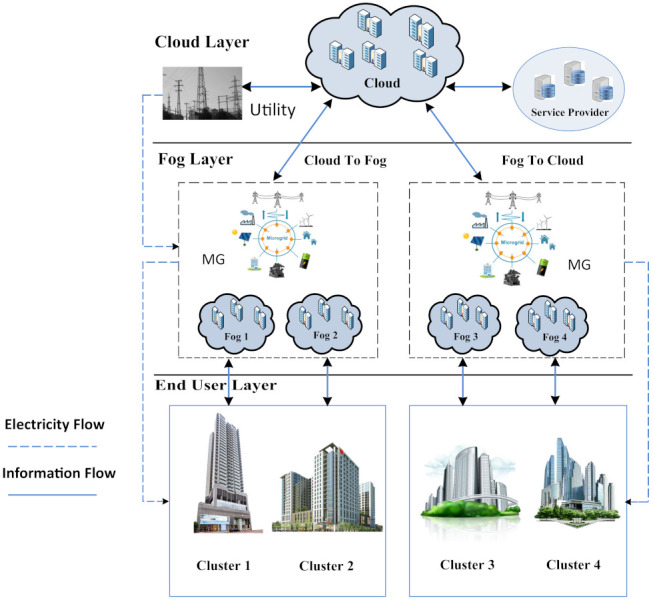
Abstract level view of proposed system model.

The model was adopted from our previous work [18,92]. Cloud service companies often have numerous data centres (dedicated to computing and storage) spread throughout the globe. Multiple fog data centres and a cloud data centres were included in the proposed system model of a cloud–fog environment. Figure 2 shows the SG framework, which is built on a geo-distributed cloud–fog environment. The end-user layer, fog layer, and core cloud layer are the three layers that make up the suggested model.

Fog includes various hardware and software resources. Fog acts as a middle layer between users and the Cloud. The Fog’s responsibility is to manage user requests that come from the different regions of the world. The requests include electricity demand and information access. The Fog is introduced to reduce the load on the Cloud. In this section, a system model with a three-layer architecture, i.e., a cloud layer, consumer layer, and a fog layer is proposed. These three layers are interlinked with each other to share information.

We suppose that the end-user layer is made up of N buildings, each with several homes. To meet the electricity demand, every residence has a renewable energy-producing unit and an energy storage system (ESS). Because it collects energy from natural sources, this sort of generator produces no pollution or fuel costs and is ecologically benign. In addition, additional generated energy is stored in the ESS during low-generation hours to meet the home’s load demand. The fog layer receives all the information regarding a home’s energy usage, generation, and appliance scheduling. To operate their applications, this layer uses a variety of cloud services. Smart metres connect the fog devices to the designated smart buildings or residences. Through the cloud–fog environment, all of the households communicate their power deficiency and excess information with one another. Smart metres communicate with one another through a local area network, a wide area network, or a metropolitan area network. In the SG, wireless communication systems such as Wi-Fi, Z-Wave, and ZigBee are available.

Fog is a cloud extension that handles user requests and works as a middleman between the client and the Cloud. The Fog connects each of the clusters. Before being transferred to the Cloud to be permanently kept, the data are briefly stored in the fog layer. Fog is more user-friendly than the Cloud and offers the same features. End users benefit from low-latency services in this way. Users’ requests are processed by virtual machines in the Fog. By submitting a request to the Fog, which is linked to MGs, users may request energy from MGs.

The fog layer, which is utilised to efficiently regulate latency and network resource management, is the second layer. The fog layer physically occurs in the clients’ local location since it is closer to them (i.e., region 1, region 2, etc.). The fog node is closer to the consumer (i.e., one hop away) when physical and communication distances are equal. Internet service providers are in charge of these fogs. The fog layer is made up of a variety of fogs. Each smart building is linked to a fog device in this manner. Fog devices are made up of virtualised hardware (H/W) resources (such as main memory, storage, network bandwidth, and CPU). Using the virtualisation idea, a virtual machine monitor manages a large number of virtual machines on a single physical computer. Many operating systems (OSs) can run simultaneously on a single H/W platform thanks to the VMM. The virtual machine manager (VMM) or hypervisor (VMWare, Xen, UML, etc.) acts as an interface between virtual machines and guest operating systems. Each virtual machine (VM) or guest operating system (guest OS), which is the central processing unit for running an application or a request, runs a variety of programmes. Fog is a communication layer that connects the user to the Cloud. The core cloud layer is the last layer. The data centres are the most significant components in this tier, since they provide storage and processing power to end users. They function on a pay-as-you-go basis, based on the application’s requirements.

Remote servers make up the cloud layer which provides on-demand data processing and administration. Clouds and fogs are inextricably connected. Fogs briefly store user data before sending the request to the Cloud for permanent storage.

**Figure 3:**
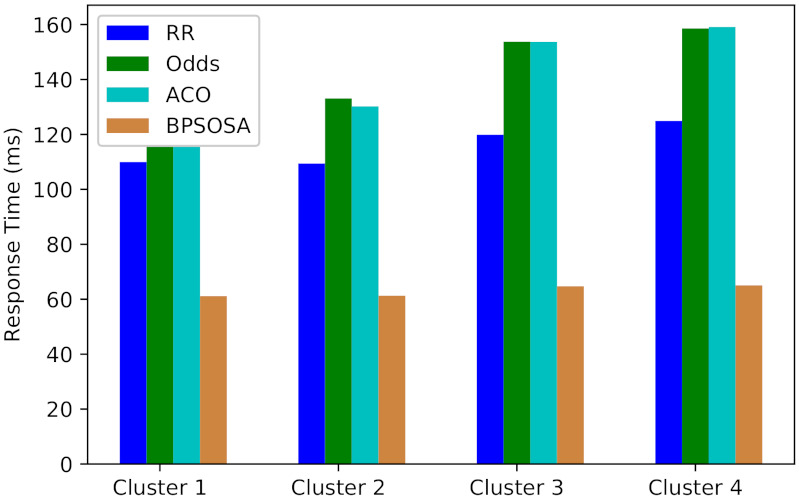
Average response time.

The computational load profile aspects of computing applications are the most essential part of cloud computing (CC). When a large number of apps are run on the same platform, the server becomes overburdened. A number of approaches were used to address this problem. This notion is shown in Figure 3 which shows how end-users produce a significant number of requests to visit the service provider. A load balancer is used in virtual machines to achieve effective load balancing and resource utilisation. CC uses a number of load-balancing strategies to provide good computational load profile control. In SGs, the computational load profile is similar to the electricity load profile concept. Thus, when we integrate the SG with the cloud–fog-based environment, then the efficient management of the computational load profile of all SG-related tasks is also necessary as well. The load-balancing problem was solved using four heuristic solutions in this study. When this system model is applied to all parts of the globe, each area has a different quantity of buildings and fogs. These structures may be found in many different settings, including residential, commercial, and industrial. Two performance evaluation alternatives are included in this system model which will be addressed in further depth in the simulation section.

There are six regions in this globe. Because these regions have a dense population and load balance difficulties, regions 2 and 3, i.e., Europe and Asia, are assumed in this research. Each region has two fogs which are linked to a cluster of structures.

Here is an overview of the strategies utilised to create excellent load balancing. The term round robin (RR) refers to a time-slicing technique that divides time into equal chunks. An equal time slicing approach is used to ensure that resources are equitably allocated across all hosts. The burden of requests from end users is distributed and balanced across virtual machines using this technology. The odds-algorithm is a mathematical approach for solving a subset of optimum halting problems. Their solution is determined by the odds strategy, and its optimality is determined by the odds importance strategy. These algorithms, on the other hand, work in a sequential fashion and do not take local or global optimal results into account. The ant colony optimisation (ACO) method is a heuristic that simulates natural behaviour using agents (ants). Ants evaluate the quality of paths based on a number of parameters (distance, amount of pheromone on the trails, etc.) and choose one at random (the better path quality, the higher probability it represents). After travelling the whole trail from source to destination, ants deposit a pheromone coating on the track to teach one another. The agent’s solution choice determines how much pheromone is left: the better the solution, the more pheromone is left. The BPSOSA method is proposed to tackle the problems in the above algorithms. This enables us to obtain the greatest results locally and globally. The current best (local best) value is compared to the previous best (global best) value in each iteration of this procedure, and the current best is chosen as the global best if it fulfils the fitness requirement. Otherwise, the first option is still the most cost-effective. BPSOSA is explained in detail below.

### 3.1. Problem Formulation

The suggested system architecture is made up of three layers. Figure 2 depicts an abstract level perspective of the suggested system model. The cloud layer is the first and highest layer. The fog layer is the second and only intermediate layer. The user layer is the third and final layer. These layers communicate with one another in order to satisfy the needs of the users. Six widely separated areas make up the suggested system model. For computations and other needed actions, the user sends requests to the Fog through the smart grid (SG). The Fog satisfies the consumers’ requests by making effective use of the resources. The task set *T* may be written as follows:

(1) $$T=T_{1},T_{2},...,T_{m}$$

The number of virtual machines in a fog can be specified as

(2) $$VM=\sum_{i=1}^{v}\left(VM_{i}\right)$$

The objective function is to minimise the processing and response time which can be formalised as

(3) $$K_{minimize}=\sum_{j=1}^{m}\sum_{i=1}^{n}\left(RT*P_{ij}*Delay\right)$$

The Fitness function is calculated as

(4) $$Fitness=Max[EXC_{vm_{1}}\left(F_{1}\right),...,EXC_{vm_{n}}\left(F_{m}\right)]$$

Here, $EXC_{vm_{1}}\left(F_{1}\right)$ is the execution time of running the set of tasks on fog $F_{1}$ on $vm_{1}$. $F_{1}$ is the set of clusters of users, i.e., $F_{1}=\left[C_{1},C_{2},...C_{x}\right]$, where x is the number of users on fog $F_{1}$. Furthermore, n is the number of VMs and m is the number of fogs.

#### 3.1.1. Processing Time

The processing time depends on the capacity of VM and length of the task. It can be calculated as follows:

(5) $$PT=\sum_{i=1}^{N}\sum_{j=1}^{M}\left(P_{ij}*A_{i}\right)$$

#### 3.1.2. Response Time

The response time is calculated as the difference between the time at which the execution of the task started and the time at which the user sent a request to be processed:

(6) RT=DelayTime+FinishTime−ArrivalTime

#### 3.1.3. Cost

Another very important factor in Cloud and Fog is cost. Data transfer cost and virtual machine cost are the two factors on which cost depends. These can be calculated using the following equations. Here, *U* is a constant factor and β is a per GB transfer cost:

(7) $$Cost_{Total}=CostDT+CostMG+CostVM$$

(8) $$CostVM=\sum_{i=1}^{N}(VMFinalTime-VMInitialTime)*U$$

(9) $$CostDT=\frac{TTotal}{DataUsed*\beta }$$

Equation (10) shows the total time taken by VM:

(10) TotalTime=FinishTime−StartTime

### 3.2. Proposed Approach

We proposed a hybrid binary particle swarm optimisation with simulated annealing (BPSOSA). In BPSOSA, the value of inertia weight is set by simulated annealing. PSO is a swarm intelligence and mobility-based resilient stochastic optimisation approach. PSO employs the concept of social interaction when it comes to problem solving. It was founded by James Kennedy and Russell Eberhart in 1995. It makes use of a swarm of agents (particles) that move around in the search space looking for the best solution. Each particle is viewed as a point in an N-dimensional space that alters its "flying" based on its own and other particles’ prior flying experiences.

PSO is a load balancing approach that is self-adaptive and meta-heuristic. The population, also known as swarm, and the solutions, sometimes known as particles, are the two most important components of PSO algorithms. The algorithms’ performance is influenced by the local best position (${\hat{p}}_{i}$) and global best position ($\hat{g}$). The fitness function generates a single value for each particle, which is referred to as the fitness value. Each particle’s objective functions are developed and validated over time. The method also provides the velocity ($v_{i}$) and location of the particle ($p_{i}$). A particle is defined as a point in D-dimensional space in this method. Each element has upper and lower limits within which it can take a value. Each particle has a D-dimensional velocity with a restricted continuous value for each element. Alternatively, each member of the velocity matrix can have a value in the range [0, 1] and the components of the binary PSO’s locations matrix can have a binary value of 0 or 1.

The Equations (11)–(15), where ω is the inertial constant, $c_{1}$ and $c_{2}$ are cognitive and social constants that are typically ∼2, and $r_{1}$ and $r_{2}$ are random numbers, represent the PSO working over a continuous space. mRange and xRange are the lowest and maximum transmission ranges, respectively, and Ran is a random number between 0 and xRange. The velocity of particle *p*’s *i*th component is updated using Equations (11)–(13), while the position of that component is updated using Equations (14) and (15):

(11) $$v_{i}=\omega v_{i}+c_{1}r_{1}\left({\hat{p}}_{i}-p_{i}\right)+c_{2}r_{2}\left(\hat{g}-p_{i}\right)$$

(12) $$\delta =\frac{xRange-mRange}{2}$$

(13) $$v_{i}=\left\{\begin{array}{cc} mRange & \mathrm{If}\;v_{i}<\delta \\ \delta & Otherwise \end{array}\right.$$

(14) $$p_{i}=Ran+v_{i}$$

(15) $$v_{i}=\left\{\begin{array}{cc} mRange & \mathrm{If}\;p_{i}<mRange\\ xRange & \mathrm{If}\;p_{i}>xRange\\ p_{i} & \mathrm{If}\;mRange\leq p_{i}\leq xRange \end{array}\right.$$

The continuous PSO equations are modified to have a binary output of 0 or 1 instead of a continuous value. The mRange and xRange boundaries are changed to 0 and 1, respectively, and replacing the Equations (12) and (13) with Equation (16) is going to give the velocities a value in the range of [0, 1]. The positions’ matrix, however, is updated using different equations than the continuous PSO as in Equations (17) and (18), where ran is any random binary and s(pi) is the particle’s sigmoid function value, i.e., Euler’s number. In the equation, the sigmoid function was utilised to scale the value to keep it inside the range [0, 1]:

(16) $$v_{i}=\left\{\begin{array}{cc} mRange & \mathrm{If}\;v_{i}<mRange\\ xRange & \mathrm{If}\;v_{i}>xRange\\ v_{i} & \mathrm{If}\;mRange\leq p_{i}\leq xRange \end{array}\right.$$

(17) $$s\left(p_{i}\right)=\frac{1}{1+e^{-p_{i}}}$$

(18) $$p_{i}=\left\{\begin{array}{cc} mRange & \mathrm{If}\;s(p_{i})\leq Ran\\ xRange & otherwise \end{array}\right.$$

ω in Equation (11) is the most important parameter as it sets the size of the search space. The search space should not be so big that it is computationally exhaustive, nor should it be so narrow that it requires too many iterations to discover the best answer. The value of omega is usually between 0.9 and 0.1. The RIW technique provided by [93] outperforms the others in terms of changing the balance between the particle’s local and global search capacities. RIW uses LDIW in addition to an SA mechanism to enhance the likelihood of obtaining a near-optimal solution with less iterations and computation time. Eberhart et al. [94] proposed LDIW in Equation (19) to reduce the negative impact of utilising the FIW technique; however, LDIW still has drawbacks, mostly due to the limited local search capacity at the start of the PSO rounds:

(19) $$\omega_{itr}^{i}=\frac{\left(\omega_{max}-\omega_{min}\right)\left(itr_{max}-itr\right)}{itr_{max}}$$

where $\omega_{itr}^{i}$ is the inertia weight of particle *i* in iteration number itr, $\omega_{max}$ is a predefined maximum possible value of inertia weight, $\omega_{min}$ is a predefined minimum possible value of ω, and $itr_{max}$ is the predefined number of maximum iterations. In our study, $itr_{max}$ is set to 500.

The complete binary particle swarm optimization is explained in the Algorithm 1 below.
**Algorithm 1:** Binary particle swarm optimisation1:calculate execution times2:initialize the swarm3:set global best4:**for**i←0→numberofiterations**do**5: **for** j←0→numberofiterations **do**6:  calculate inertia value7:  calculate new velocities8:  calculate new positions9:  calculate fitness value10:  evaluate solution11:  update particle memory12:  update global best13: **end for**14:**end for**

Even if a particle starts at the LDIW global optimisation point, it will quickly move away from it. Similarly, if a particle does not discover a near-optimal solution and remains stuck in one sector of space, its global search ability reduces due to the linear drop in ω, reducing the chances of finding a better solution. As a result, the iteration forepart’s capacity to find the closest ideal solution improves. An SA technique was utilised in combination with the LDIW to solve the LDIW issues.

The main idea behind RIW, as discussed before, is to overcome the negative influences of LDIW on both local and global search abilities. To achieve this, RIW learns from historical velocities and the fitness of the particle where ω make the historical effect by randomly selecting inertia weights and later adaptively adjusts with the best solution found. To learn from the historical velocities and fitness values, Yue-lin et al. [93] used an annealing method with a cooling temperature function, as shown in Equation (20). To increase the probability of changing the particle’s speed, the average fitness values of each particle along with the best fitness recorded by any particle are used in the equation:

(20) $$cTemp_{itr}=\left(\frac{pFitness_{avg}^{itr}}{pFitness_{best}}\right)-1$$

In this equation, $cTemp_{itr}$ is the annealing temperature value in the current iteration itr, $pFitness_{avg}^{itr}$ is the average of all recorded fitness throughout all iterations until the current iteration, and $pFitness_{best}$ is the best recorded fitness of the particle.

According to the aforementioned annealing temperature in Equation (20), ω will be adjusted according to Equation (22), which is the annealing probability of the proposed method and will be calculated according to Equation (23), where ρ is the annealing probability, $pFitness^{itr}$ is the particle current fitness in current iteration itr, $pFitness^{itr-k}$ is the previous particle’s fitness in iteration itr−k; where *k* is a fixed number, *e* is Euler’s number, $cTemp_{itr}$ is the cooling temperature from Equation (20), $\omega_{itr}^{i}$ is the inertia weight ω of particle *i* in iteration number itr, and ran is any binary random number:

(21) $$\eta =\frac{pFitness^{itr-k}-pFitness^{itr}}{cTemp_{itr}}$$

(22) $$\rho_{i}^{itr}=\left\{\begin{array}{cc} 1; & pFitness^{itr-k}\leq pFitness^{itr}\\ e^{-\eta }; & Otherwise \end{array}\right.$$

(23) $$\omega_{itr}^{i}=\left\{\begin{array}{cc} 1+\frac{ran}{2}; & \rho \geq ran\\ 0+\frac{ran}{2}; & Otherwise \end{array}\right.$$

The Algorithm 2 below explains the complete method to calculate inertia weight (ω) using simulated annealing.
**Algorithm 2:** Calculate inertia weight (ω) using the simulated annealing method1:define value of *k*2:define $\omega_{max}$ = 0.93:define $\omega_{min}$ = 0.14:**if**itr is a multiple of *k* **then**5: **if** $pFitness^{itr-k}\leq pFitness^{itr}$ **then**6:  $\rho_{i}^{itr}=1$7: **else**8:  $cTemp_{itr}=\left(\frac{pFitness_{avg}^{itr}}{pFitness_{best}}\right)-1$9:  $\eta =\frac{pFitness^{itr-k}-pFitness^{itr}}{cTemp_{itr}}$10:  $\rho_{i}^{itr}=e^{-\eta }$11: **end if**12: **if** ρ≥ran **then**13:  $\omega_{itr}^{i}=1+\frac{ran}{2}$14: **else**15:  $\omega_{itr}^{i}=0+\frac{ran}{2}$16: **end if**17:**end if**18:**if**itr is not a multiple of *k* **then**19:  calculate $\omega_{itr}^{i}=\frac{\left(\omega_{max}-\omega_{min}\right)\left(itr_{max}-itr\right)}{itr_{max}}$20:**end if**

## 4. Simulations and Discussion

Using Java Netbeans, the experiment is run in the CloudSim simulator. The method may also be incorporated into CloudAnalyst, a CloudSim toolkit extension. CloudAnalyst also has a graphical user interface. CloudSim employs virtual machines with a variety of hardware characteristics.

### 4.1. Response Time Summary

The response time for clusters of users using round robin, odds algorithm, ACO, and BPSOSA algorithms is shown in Figure 3. In Figure 3, we can see that with the round robin, odds algorithm, and ACO, the response time is much higher compared to the BPSOSA. The response time was optimised for all the clusters of users simultaneously. For Cluster 1, the average response time obtained by the round robin, odds algorithm, ACO, and BPSOSA is 109.95 ms, 131.29 ms, 131.70 ms, and 61.12 ms, respectively. In this case, BPSOSA outperforms the round robin by 48.83 ms, the odds algorithm by 70.17 ms, and ACO by 70.58 ms. For Cluster 2, the average response time obtained by the round robin, odds algorithm, ACO, and BPSOSA is 109.43 ms, 133.08 ms, 130.20 ms, and 61.28 ms, respectively. In this case, BPSOSA outperforms the round robin by 48.15 ms, the odds algorithm by 71.28 ms, and ACO by 68.92 ms. For Cluster 3, the average response time obtained by the round robin, odds algorithm, ACO, and BPSOSA is 119.87 ms, 153.74 ms, 153.70 ms, and 64.69 ms, respectively. In this case, BPSOSA outperforms the round robin by 55.18 ms, the odds algorithm by 89.05 ms, and ACO by 89.01 ms. For Cluster 4, the average response time obtained by the round robin, odds algorithm, ACO, and BPSOSA is 124.90 ms, 158.57 ms, 159.11 ms, and 65.05 ms, respectively. BPSOSA outperforms the round robin by 59.85 ms, the odds algorithm by 93.52 ms, and ACO by 94.06 ms.

By efficiently allocating resources, BPSOSA reduces the burden on the Fog. Rather than utilising a random ω or a linearly declining ω, BPSOSA utilises a simulated annealing approach to discover the best feasible solution for each job and schedules requests in the most efficient way. When a job comes, the load balancer calculates the virtual machine’s memory, usage, power consumption, and speed. Furthermore, depending on these variables, the job to be processed is assigned to the virtual machine with the greatest priority, ensuring that the process does not have to wait long. When compared to the round robin, odds algorithm, and ant colony optimisation, the results demonstrate that it is significantly superior for load balancing. The results demonstrate that the odds method and ant colony optimisation are quite similar. Both the odds method and ant colony optimisation performed worse than the round robin. The total overview of the response times for the methods discussed above is shown in Table 1. Other algorithms are outperformed by BPSOSA.

**Figure 4:**
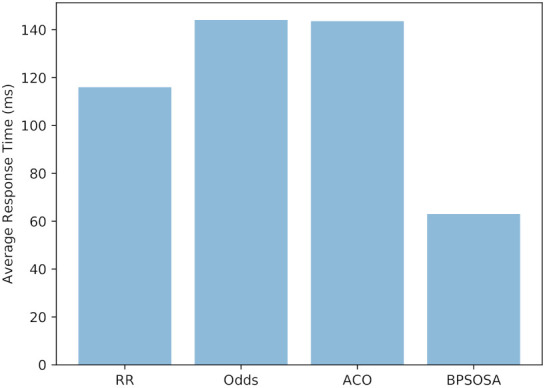
Average response time of clusters.

The round robin, odds algorithm, ACO, and BPSOSA have average reaction times of 116.01 ms, 144.10 ms, 143.60 ms, and 63.02 ms, respectively, as shown in Figure 4.

### 4.2. Processing Time Summary

**Figure 5:**
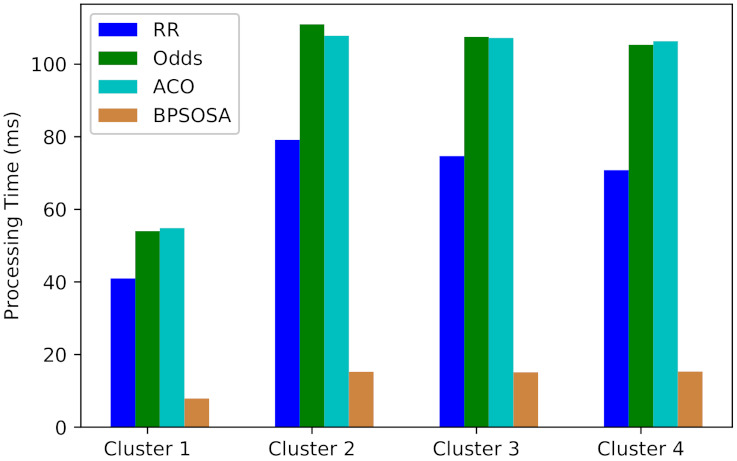
Processing time comparison of different algorithms.

The processing time for clusters of users using the round robin, odds algorithm, ACO, and BPSOSA algorithms are shown in Figure 5. In Figure 5, we can see that with the round robin, odds algorithm, and ACO, the processing time is much higher as compared to the BPSOSA. The processing time was optimised for all the clusters of users simultaneously. For Cluster 1, the average processing time obtained by the round robin, odds algorithm, ACO, and BPSOSA is 40.98 ms, 54.00 ms, 54.85 ms, and 7.89 ms, respectively. BPSOSA outperforms the round robin by 33.09 ms, the odds algorithm by 46.11 ms, and ACO by 46.96 ms. For Cluster 2, the average processing time obtained by the round robin, odds algorithm, ACO, and BPSOSA is 79.16 ms, 110.97 ms, 107.83 ms, and 15.25 ms, respectively. In this case, BPSOSA outperforms the round robin by 63.91 ms, the odds algorithm by 95.72 ms, and ACO by 92.58 ms. For Cluster 3, the average processing time obtained by the round robin, odds algorithm, ACO, and BPSOSA is 74.69 ms, 107.51 ms, 107.22 ms, and 15.12 ms, respectively. In this case, BPSOSA outperforms the round robin by 59.57 ms, the odds algorithm by 92.39 ms, and ACO by 91.21 ms. For Cluster 4, the average processing time obtained by the round robin, odds algorithm, ACO, and BPSOSA is 70.82 ms, 105.53 ms, 106.33 ms, and 15.32 ms, respectively. In this case, BPSOSA outperforms the round robin by 55.50 ms, the odds algorithm by 90.21 ms, and ACO by 106.33 ms respectively.

BPSOSA optimises the load on the Fog with the help of multiple agents which are called particles. These particles search for an optimal solution with the help of its deterministic and stochastic components. Another reason for its better performance is that, unlike other optimisation methods, it has less coefficients to be tuned. In every iteration, a particle moves closer to the optimal solution. The binary implementation of PSO helps to avoid overloading of a single processor, that is the case with several other methods. The simulated annealing method to adjust the inertia weight in every iteration also helps find the optimal solution in a faster and more efficient manner. The results also support this argument.

**Figure 6:**
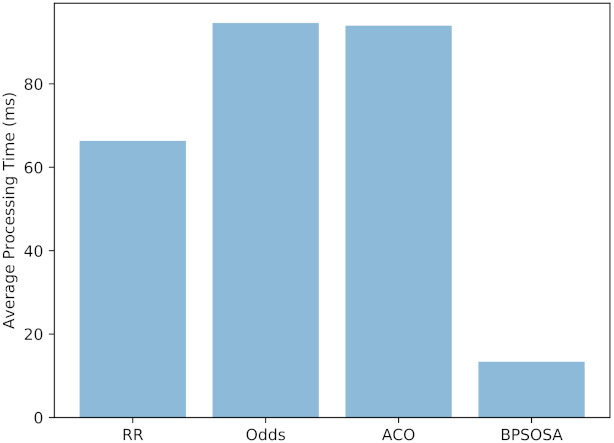
Average processing time of fogs.

Figure 6 and Table 2 shows the average processing time obtained by the round robin, odds algorithm, ACO, and BPSOSA is 66.33 ms, 94.59 ms, 93.95 ms, and 13.39 ms, respectively.

### 4.3. Cost Summary

**Figure 7:**
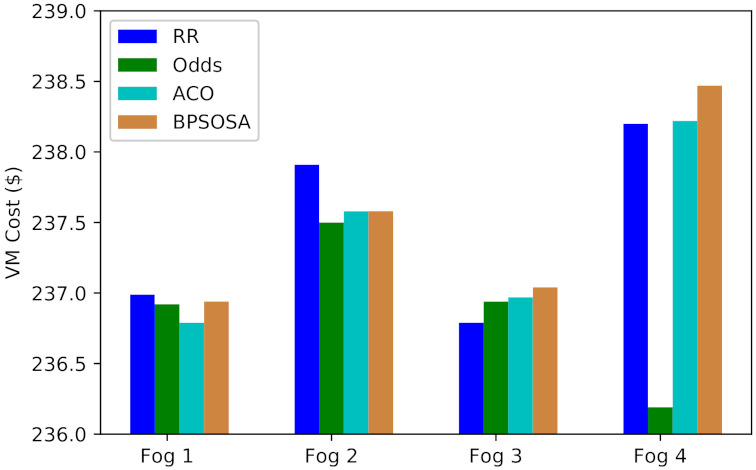
Average virtual machine cost of fogs.

**Figure 8:**
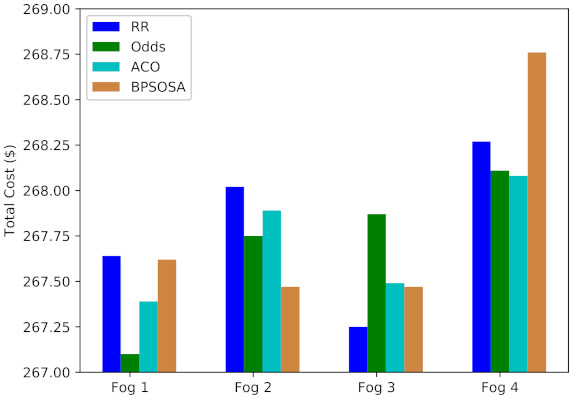
Average total cost of fogs.

Figure 7 shows the VM cost and Figure 8 shows the VM cost for the clusters of users using the round robin, odds algorithm, ACO, and BPSOSA algorithms. In Figure 8, we can see that with the round robin, odds algorithm, and BPSOSA, the cost is higher than with ACO. The cost was optimised for all the clusters of users simultaneously.

In terms of cost, the results demonstrate that ACO is a superior load-balancing algorithm than round robin, odds algorithm, and BPSOSO. The results demonstrate that the odds method and ant colony optimisation are quite similar. Both the round robin and ant colony optimisation were outperformed by the odds method. The overall overview of the cost summary of the previously stated algorithms is shown in Table 3.

**Figure 9:**
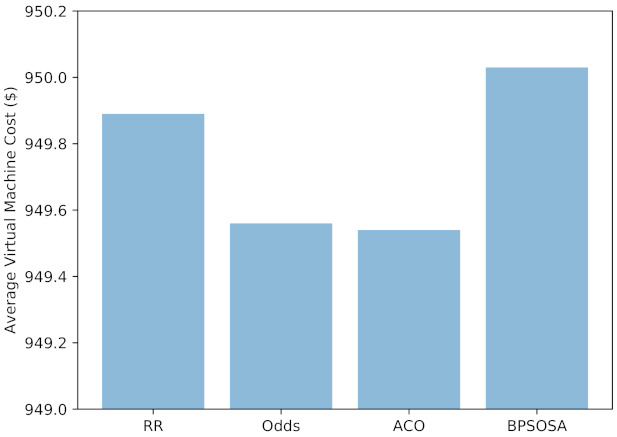
Average virtual machine cost.

Figure 9 shows the average VM cost obtained by round robin, odds algorithm, ACO, and BPSOSA is USD 949.89, USD 949.54, USD 949.46, and USD 950.03, respectively.

**Figure 10:**
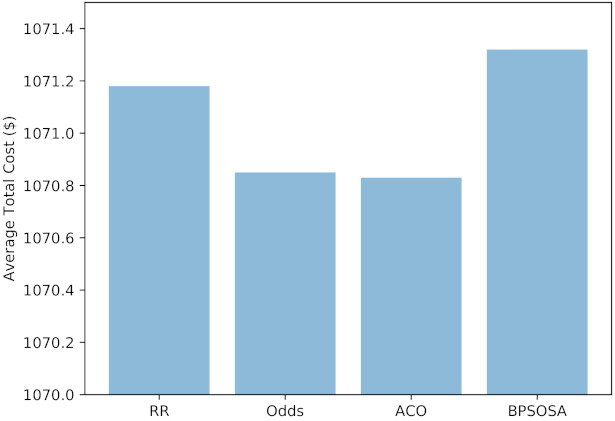
Average cost.

Figure 10 shows the average cost obtained by round robin, odds algorithm, ACO, and BPSOSA is USD 1071.89, USD 1070.85, USD 1070.83, and USD 1071.32, respectively.

We may deduce from the simulation results that the suggested algorithm BPSOSA outperforms the round robin, ACO, and the odds algorithm. The higher performance of BPSOSA is due to a combination of the best characteristics of the PSO combined with the inertia weight modified by simulated annealing. BPSOSA and ACO provided the best global and local solutions, respectively. Due to its sluggish convergence, the ACO’s response time, processing time, execution time, and cost were somewhat greater. ACO may become caught in local optima, preventing it from finding the global best solution.

To address the concerns with ACO in the future, the ABC fitness step might be incorporated into the algorithm, which would result in a faster reaction time, processing time, cost, and execution time due to its greater convergence rate.

## 5. Conclusions

A system model of cloud- and fog-based environment combined with SG is provided in this study. This model is made up of three layers: a cloud layer, a fog layer, and an end-user layer. Cloud servers are put in the cloud layer, fog servers and virtual machines are deployed in the fog layer, and the end-user layer is made up of a cluster of buildings, each with several residences. We presented a new method based on binary particle swarm optimisation with inertia weight adjustment via simulated annealing. The technique is named BPSOSA. The inertia weight is an important factor in BPSOSA which adjusts the size of the search space for finding the optimal solution. BPSOSA is evaluated against the round robin, odds algorithm, and ant colony optimisation. In terms of response time, BPSOSA outperforms the round robin, odds algorithm, and ant colony optimisation by 53.99 ms, 82.08 ms, and 81.58 ms, respectively. In terms of processing time, BPSOSA outperforms the round robin, odds algorithm, and ant colony optimisation by 52.94 ms, 81.20 ms, and 80.56 ms, respectively. Ant colony optimisation has a slightly better cost efficiency but the difference is insignificant.

In the future, our research will look at this prescribed approach in the business sector. It will also be enhanced to allow users to control a range of load-balancing programmes. That is, among other things, to improve the SG efficiency, appliance scheduling, and micro-grid generation. In the future, rather than simulations, the proposed BPSOSA technology will be compared to other artificial intelligence-based technologies under real-time circumstances.

## Figures and Tables

**Table 1:** Overall response time summary of all algorithms.

Algorithms	Average (ms)	Minimum (ms)	Maximum (ms)
Round Robin	116.01	41.79	584.69
Odds Algorithm	144.10	42.63	581.35
ACO	143.60	42.63	577.13
BPSOSA	63.02	38.70	87.37

**Table 2:** Overall processing time summary of all algorithms.

Algorithms	Average (ms)	Minimum (ms)	Maximum (ms)
Round Robin	66.33	0.30	531.56
Odds Algorithm	94.59	0.64	530.53
ACO	93.95	1.14	530.31
BPSOSA	13.39	0.17	26.12

**Table 3:** Overall cost summary of all algorithms.

Algorithm	VM Cost (USD)	Data Transfer Cost (USD)	Total Cost (USD)
Round Robin	949.89	121.29	1071.18
Odds Algorithm	949.56	121.29	1070.85
ACO	949.54	121.29	1070.83
BPSOSA	950.03	121.29	1071.32

## Data Availability

Not applicable.

## Abbreviations

The following abbreviations are used in this manuscript:

SG	Smart Grid
IoT	Internet of Things
VM	Virtual Machine
PaaS	Platform as a Service
SaaS	Software as a Service
IaaS	Infrastructure as a Service
MG	Micro-Grid
CI	Computational Intelligence
SA	Simulated Annealing
BPSO	Binary Particle Swarm Optimisation
NIST	National Institute of Standards and Technology
RFID	Radio Frequency Identification
WSN	Wireless Sensor Networks
RES	Renewable Energy Source
SOM	Service-Oriented Middleware
BPSOSA	Binary Particle Swarm Optimisation Simulated Annealing
